# Unmet Need for Modern Contraceptive Methods Among Displaced Married Women in Their Reproductive Years in Bishan Guracha Town, West Arsi Zone, Oromia Region, Ethiopia

**DOI:** 10.1155/2024/6662117

**Published:** 2024-09-18

**Authors:** Sisay Million, Zeleke Gebru, Sultan Hassen, Selamnesh Tesfaye

**Affiliations:** ^1^ School of Public Health Hawassa University Comprehensive Specialized Hospital, Hawassa, Ethiopia; ^2^ School of Public Health College of Medicine & Health Sciences Arba Minch University, Arba Minch, Ethiopia; ^3^ Department of Health Extension Arba Minch College of Health Science, Arba Minch, Ethiopia

**Keywords:** Ethiopia, internally displaced, modern contraceptive methods, unmet need

## Abstract

**Background:** Refugees and conflict-affected areas are often unreached by national strategies and programs. As a result, high unmet needs are more likely because of their social interruption with their traditional information sources, support, protection, and lack of income which limits refugees' ability to make a free choice that would allow them to plan and space the number of children they desire. Information on the unmet needs of internally displaced persons (IDPs) women is scarce. This study is aimed at assessing the magnitude of the unmet need for modern contraceptive methods and associated factors among IDPs currently married reproductive-age women.

**Methods:** A community-based cross-sectional study was conducted among 393 internally displaced women currently married reproductive-age women using a simple random sampling method using a structured, pretested, and interview-administered questionnaire. A logistic regression model was used to identify associated factors. Statistically significant variables at *p* value < 0.25 in the bivariate analysis were entered into multivariable analysis, and statistical significance was declared at *p* value ≤ 0.05.

**Results:** About 160 (40.7%) (95% CI: 35.94%–45.67%) of women had an unmet need for modern contraceptive methods, 139 (35.4%) for spacing, and 21 (5.3%) for limiting. Less than 18 years of age at first marriage, lack of access to modern contraception, lack of discussion with healthcare providers, and travel time of 30 min or more to obtain family planning were found to be risk factors for unmet contraceptive needs. The risk of unmet need for modern contraceptives was high among women who were married at age of less than 18 years of age in comparison with women who were married at 18 and above (AOR = 1.559; 95%CI = 1.019–2.385). Unmet needs were higher among participants who had no adequate availability of modern contraceptive methods than those who had adequate availability of modern contraceptive methods (AOR = 1.738; 95%CI = 1.125–2.684). Similarly, the odds of unmet needs were 1.673 times higher among participants who did not discuss FP with healthcare providers than those who discussed FP with healthcare providers (AOR = 1.673; 95%CI = 1.085–2.581). Moreover, the odds of unmet needs were 1.551 times higher among participants who traveled 30 min and above to access family planning services as compared to those respondents who traveled below 30 min (AOR = 1.551; 95%CI = 1.002–2.401).

**Conclusion and Recommendations:** The magnitude of the unmet need for modern contraceptive methods was higher than both the Ethiopian national and Oromia regional state total unmet need for the general population. Governmental and nongovernmental organizations should increase their efforts to reduce this high magnitude of unmet needs by emphasizing those factors that have a great contribution to unmet needs.

## 1. Introduction

Family planning (FP) is the ability of individuals and couples to anticipate and attain their desired number of children and the spacing and limiting of their birth, which is realized through the use of contraceptive methods [1]. Modern contraceptive methods are defined as products or medical procedures that inhibit reproduction from acts of sexual intercourse [2]. Unmet need for contraception is the proportion of currently married women or in a sexual union avid to limit or space childbirth but not using any contraceptive methods. The idea of unmet need shows the gap between women's reproductive intentions and their contraceptive practice [3].

Ethiopia is the second-biggest refugee-hosting country in Africa; the United Nations High Commissioner for Refugees estimated that the country had 758,199 registered refugees [4]. Ethiopia recorded the third maximum number of new displacements worldwide, with 3,191,000 internally displaced persons (IDPs) recognized. A big percentage of these displacements are warfare-induced, basically related to ethnic and border-based clashes [5].

Globally, in displaced settings, an estimated 26 million women and girl refugees are affected extremely by emergencies and face various sexual and reproductive health risks, requiring access to crucial services, including contraception [6]. The percentage of using a modern contraceptive method was 24% among married and all age groups of Syrian refugee women in Turkey. The proportion of unmet FP needs was about 35%. Overall, the uptake of FP during refuge and displacement is often compromised [7]. Many women and couples want to space or limit pregnancies following displacement. Across diverse contexts, 30%–40% of women experiencing displacement did not want to become pregnant in the next 2 years, and 12%–35% wanted to limit the number of pregnancies [8].

In a humanitarian setting in northern Uganda, the current use of any modern FP method increased from 7.1% to 22.6%, and long-acting and permanent methods increased from 1.2% to 9.8% [9]. Ladies with a neglected requirement for FP were twice as prone to have had a spontaneous pregnancy during their reproductive life compared with ladies with met needs. A high pace of unplanned pregnancy is generally related to a high pace of intentionally initiated fetus removals [10].

Overall, 43% of accidental pregnancies happened in creating a world, and 74% of them were identified with neglected requirements for FP. While in East Africa, the neglected requirement for family arranging is answerable for 86% of accidental pregnancies [7]. In outcast camps and uprooted settings, ladies and kids are exposed to assault, accidental pregnancy, risky fetus removals, and sexually transmitted infection (STI)/human immunodeficiency virus (HIV) [11]. In the Democratic Republic of Congo (DRC), among internally displaced women, more than 40% of women reported having experienced an unintended pregnancy, and more than 20% reported a history of induced abortion [8].

“Marginalized populations such as refugees, IDPs, and migrants, regularly unreached through national strategies and programs, face barriers to information and access that would permit them to plan and space the number of children they need. Focusing on family planning in this population is crucial in preventing the social, economic, and health outcomes resulting from unintended pregnancies” [12].

“Many humanitarian investment necessities did not include contraception, and where family planning was provided, women had very little access to the highly effective, long-acting methods they wanted and needed. Emergency birth control becomes unavailable out of doors post-rape care. Consequently, many women and girls are pressured to deal with unwanted pregnancies and experience preventable morbidity and mortality from complications of pregnancy and childbirth, in addition to the hardship of conflict and displacement” [13].

Even though the research on the sexual and reproductive health of conflict- and disaster-affected populations is gaining attention globally, there is scanty research on the unmet needs for modern contraception among women in refugees [14]. If all unmet needs for modern-day contraception have been fulfilled in growing areas, there might be around a three-quarters decline in unintended pregnancies (from the contemporary 89 million to 22 million in keeping with year), unplanned births (from 30 million to seven million per year), and triggered abortions (from 48 million to 12 million per year). Fully assembly the unmet need for modern contraception might bring about an envisioned 76,000 much fewer maternal deaths each year [15].

As a part of an effort to reduce the unmet need for contraception among women in refugee and IDP settings, the Interagency Working Group (IAWG) on Reproductive Health in Crises develop guidelines for providing reproductive health services in refugee settings. Essential reproductive health interventions should be implemented at the beginning of a humanitarian crisis, with a call for a multisectoral approach to address the comprehensive sexual and reproductive health needs of affected populations. Additionally, fostering coordination among partners is crucial. However, despite these efforts, the demand for modern contraceptives remains significantly unmet [16].

Currently, the level of unmet needs for modern contraceptive methods and their associated factors in this study area is unknown. Therefore, this study is aimed at assessing the magnitude and associated factors of the unmet need for modern contraceptive methods among married reproductive-age women in Bishan Guracha Town in an internally displaced setting.

## 2. Methods and Materials

### 2.1. Study Area, Period, and Study Design

The study was conducted at Bishan Guracha Town, West Arsi Zone, Oromia Region, Ethiopia, from April 1 to July 15, 2021. Bishan Guracha Town was a small town that was located in the West Arsi Zone, Oromia Region, Ethiopia. The town was situated 265 km away from Addis Ababa, the capital city of Ethiopia, and 5 km from Hawassa, the capital city of southern nations, nationalities, and people's regions. There is an IDP area located 2 km away from Bishan Guracha Town. According to a report from the administration body of the IDPs, the internally displaced area has a total population of 6490 with households of 1352, and women of reproductive age were 836. There is one newly constructed health center that was giving service to this population in an area and one primary school. These populations stayed for 3 years in an internally displaced area and are currently living under the support of Ethiopian governments. A community-based cross-sectional study design was employed.

### 2.2. Study Population

All currently married women of reproductive age in the internally displaced area in Bishan Guaracha were the source population. The study populations were selected from currently married women of reproductive age at IDPs in Bishan Guaracha. Currently married women of reproductive age who were critically ill, unable to hear, and had known mental health problems during the day of data collection were excluded.

### 2.3. Sample Size Determination

The sample size was calculated by using a single population proportion formula by considering 41.8% of total unmet needs, which was taken from the study conducted in Eritrean refugee camps, Tigray, North Ethiopia (*p* = 0.418) [17], with the level of confidence to be 95% (*α* = 0.05), *Z* *α*/2 = 1.96, and the margin of error to be 5% (*d* = 0.05), and by assuming 5% nonresponse rates, which gives a total sample size of 393.

### 2.4. Sampling Techniques and Procedure

A simple random sampling method using a table of random numbers technique was applied to select currently married women in the reproductive-age group after lists of participants were taken from health extension workers. There was strong vital registration, especially for women who recently joined marriage before the survey. Finally, the selected currently married reproductive-age woman was interviewed to participate in the study after obtaining informed consent. A mark on the house was made after interviewing the selected participant to prevent duplication or reinterviewing. If sampled women were not found or if their home was closed, three visits would be made, and after three visits, they would be recorded as nonrespondents, and data collection was done in the morning to reduce the nonresponse rate.

### 2.5. Operational Definitions and Definitions of Terms

#### 2.5.1. Unmet Need for Limiting Childbirth

The proportion of fecund, sexually active women who wish to stop childbearing completely but are not using any modern contraceptive method or who were pregnant whose current pregnancy is unwanted. It also includes women whose last birth in the past 2 years was unwanted [18].

#### 2.5.2. Unmet Need for Spacing Childbirth

The proportion of fecund, sexually active women who want another child after 2 years but are not using any modern contraceptive method, who say they are unsure whether they want another child, who want another child but are unsure when to have the child, or pregnant women whose current pregnancy was mistimed. It also includes women whose last birth in the past 2 years was mistimed [18].

#### 2.5.3. Unmet Need for Modern Contraception

Any woman who wants to limit or space childbirth but does not use modern contraceptive methods is considered to have an unmet need for modern contraception [18].

The dependent variable was an unmet need for modern contraceptive methods and dichotomized as the absence of an unmet need (0 = *no*) and having an unmet need (1 = *yes*).

#### 2.5.4. Currently Married Women

Ladies who were either officially or legally married and who were living in a consensual union [6].

#### 2.5.5. Unfavorable Attitude

A woman who had a collective sum value for six attitudinal statements response was lower than or equal to the sum median score value of all respondents [19].

#### 2.5.6. Favorable Attitude

For a woman who had a collective sum value for six attitudinal questions, the response was greater than the sum median score value of all respondents [19].

#### 2.5.7. Good Knowledge

A woman who had a collective sum value for eight knowledge statement responses was greater than the sum median score value of all respondents [19].

#### 2.5.8. Poor Knowledge

A woman who had a collective sum value for eight knowledge statement responses was less or equal to the sum median score value of all respondents [19].

Knowledge assessment question with multiple responses, the respondent who mentioned at least two from a given response (1 = *yes*) and otherwise (0 = *no*) [20].

#### 2.5.9. Refugee

A refugee is “someone who, as a result of a well-based worry of being offended for reasons of race, religion, nationality, membership of a selected social group or political opinion, is outside the country of his nationality and is unable to, or due to such fear, is unwilling to benefit himself of the security of that country” [21].

#### 2.5.10. IDPs

IDPs are human beings who have fled their homes for similar reasons to refugees; however, they have no longer crossed an international border. Instead, they remain displaced from their own country [21].

### 2.6. Data Collection Tools and Procedures

Data collection tools were adapted from EDHS and by reviewing different kinds of literature that were developed for a similar study. The questionnaire section included sociodemographic characteristics, reproductive-related, FP program-related, and behavior-related factors. After getting informed consent, face-to-face data collection was done using structured, pretested interview-administered questionnaires. Data were collected by five BSc midwives who were engaged in the provision of reproductive health services in health facilities around that area. In addition to an investigator, an experienced BSc health officer was assigned to supervise, lead, and assist data collectors.

### 2.7. Data Quality Control

To keep the quality of data, questionnaires were prepared initially in English by an investigator and translated to Afaan Oromo to avoid misinterpretation of words, then retranslated back to English by a language expert to keep its consistency. Before data collection, 1-day training was given for data collectors and supervisors. To make sure that the questionnaires developed were based on findings from other similar studies and were appropriate and understandable, a pretest was done on 5% of the sample size at Arsi Negelle Town before actual data collection to check completeness; consistency of questionnaires; understanding of the questions, wording, and logic; and skip the order of the questions in a sensible way for the respondents. Internal consistency of the items was checked by using Cronbach's alpha (*α*) for the scale measuring questionnaire of attitude, found to be (*α* = 0.827), which indicates an acceptable level of reliability.

### 2.8. Data Processing and Analysis

After checking the completeness, consistency, and clarity of the data collected by the investigator, the data were entered into EpiData Version 4.6. Then, it was exported to the Statistical Package for Social Science (SPSS) Version 25 for analysis. The results were presented in the form of tables, figures, and text using frequencies, mean standard deviation, and percentages to describe the study population about relevant variables. Bivariable logistic regression was used to model the study findings. In the bivariable logistic regression analysis, variables with *p* value < 0.25 were entered into a multivariable logistic regression analysis to see an independent association with the dependent variable and also to determine the odds ratio and 95% confidence interval. Multicollinearity was checked using a cut-off point based on variance inflation factor (VIF) < 10. In addition, a goodness-of-fit model was checked by the Hosmer–Lemeshow test. The test was insignificant (*p* value = 0.62), and the Omnibus test of the model coefficient was significant (*p* value < 0.001). Backward logistic regression was used to classify variables that had a major contribution to the model. Odds ratios with 95% CI were reported to show the direction and strength of the association. The level of significance was considered at a *p* value ≤ 0.05.

### 2.9. Ethical Considerations

Ethical clearance was obtained from the Institutional Ethical Review Board (IRB) of Arba Minch University College of Medicine and Health Science based on the ethical clearance with reference number IBR/1081/21. Written informed consent was obtained from each participant. Informed consent was obtained from the guardian among participants aged <18 years old.

## 3. Result

### 3.1. Sociodemographic Characteristics of the Study Participants

A total of 393 participants were included in the study, with a response rate of 100%. The mean age of the participants was 28 (SD ± 4.6) years old. The majority of the study participants (379, 96.4%) were Oromo in their ethnicity, and 353 (89.8%) were housewives. Almost half of the participants (189, 48.1%) attended primary education, and 209 (53.2%) of their partners also attended primary education. Additionally, the majority of the study participants (356, 90.6%) were Muslim with their religious status, and 253 (64.4%) of the study participants resided in urban areas before they were displaced (Table 1).

### 3.2. Reproductive Health Characteristics of the Study Participants

The mean age of marriage of respondents was 17 (SD ± 2.4) years old. Around half (200, 50.9%) of the respondents were in the age range of below 18 years when they got married for the first time, and 121 (30.8%) were pregnant at the time of the interview. About 89 (73.6%) of pregnancies were wanted, 30 (24.8%) wanted a later, and two (1.7%) did not want to be pregnant at all. Around 152 (38.7%) of respondents had one up to two children, and 87 (22.1%) of respondents had a history of induced abortion (Table 2).

### 3.3. FP Program and Behavioral-Related Characteristics of Study Participants

About 281 (71.5%) of respondents had ever used modern contraceptive methods. Around 178 (45.3%) of respondents had discussed FP use with their partners. Similarly, 165 (42.0%) of respondents had discussed FP with healthcare providers, and about 238 (60.6%) of respondents had no radio in their house (Table 3).

### 3.4. Knowledge and Attitude of Respondents Towards Modern Contraceptive Methods

About 380 (97%) of the study participants had ever heard of modern contraceptive methods. The most commonly known methods were pills and injectables. Around 341 (87%) of the study participants knew injectables, and 289 (73.5%) of the participants knew pills. Their major source of information was health workers and radio. About 313 (79.6%) of the respondents heard from health workers, and 280 (71.2%) heard from the radio (Table 4).

#### 3.4.1. The Magnitude of the Unmet Need for Modern Contraceptive Methods

About 160 (40.7%) (95% CI: 35.94%–45.67%) of women had an unmet need for FP, 139 (35.4%) for spacing, and 21 (5.3%) for limiting.

### 3.5. Algorithm to Estimate the Total Unmet Need

**Figure 1:**
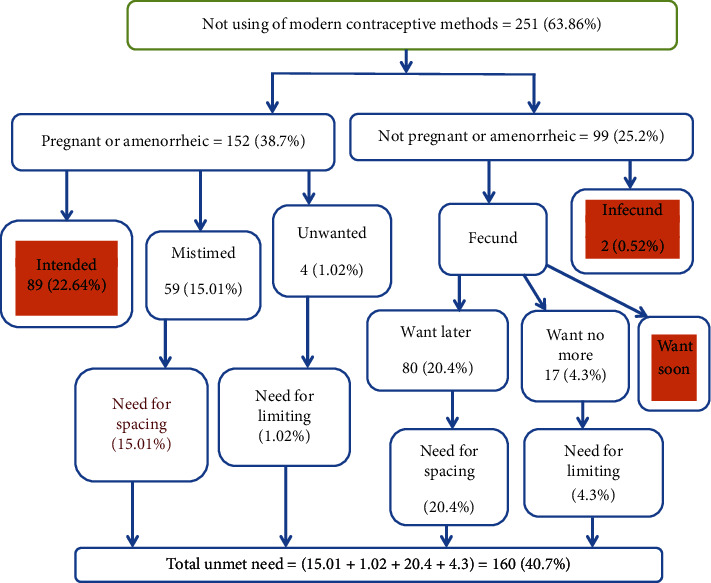
Algorithm of unmet need for modern contraceptive methods among internally displaced currently married reproductive-age group women in Bishan Guracha Town, West Arsi Zone, Oromia Region, 2021 [18]. Note: The total numbers (393) were the denominator for all the percentages.

Those women whose last birth was mistimed and unwanted and women whose current pregnancy was intended, mistimed, and unwanted were categorized under the subalgorithm of pregnant or amenorrheic. Last births mistimed and unwanted were added to women whose current pregnancies were mistimed and unwanted (Figure 1).

### 3.6. Factors Associated With Unmet Need for Modern Contraceptive Methods

In the bivariate logistic regression analysis, each explanatory variable was assessed in association with an unmet need for modern contraceptive methods. Among these factors, the mother's attitude and knowledge about FP, discussion with their partner, discussion with healthcare providers, age at first marriage, ever experienced abortion, accessibility of FP service, and availabilities of desired modern contraceptive methods were candidate variables for the final multivariable logistic regression model analysis (at *p* value < 0.25) (Table 5).

In multivariable logistic regression analysis, age at first marriage (AOR = 1.559; 95%CI = 1.019–2.385), availabilities of modern contraceptive methods (AOR = 1.738; 95%CI = 1.125–2.684), accessibility of FP service (AOR = 1.551; 95%CI = 1.002–2.401), and discussion with healthcare providers (AOR = 1.673; 95%CI = 1.085–2.581) were among factors significantly associated with unmet need for modern contraceptive methods (Table 5).

The odds of unmet need were 1.738 times higher among participants who had no adequate availability of modern contraceptive methods than those who had adequate availability of modern contraceptive methods (AOR = 1.738; 95%CI = 1.125–2.684). Similarly, the odds of unmet needs were 1.673 times higher among participants who did not discuss FP with healthcare providers than those who discussed FP with healthcare providers. Moreover, the odds of unmet needs were 1.559 times higher among participants who got married before the age of 18 years than those who got married at the age of 18 and above. The odds of unmet needs were 1.551 times higher among participants who traveled on trips 30 min and above to access FP services as compared to those respondents who traveled on a trip below 30 min (Table 5).

## 4. Discussions

The findings of this study revealed that the total magnitude of unmet need for modern contraceptive methods among married women of reproductive age was 40.7% (95%CI = 35.94%–45.67%), unmet need for spacing (35.4%), and unmet need for limiting (5.3%). This finding was higher than studies conducted in refugee settings in Canada (26.8%) and Djibouti (8.8%) [22, 23]. The inconsistency might be due to differences in access to healthcare services, availability of modern contraceptive methods, involvement of nongovernmental organizations, socioeconomic status, and educational status. This finding is also higher than the global average of 12.3% [24]. The differences might be explained by socioeconomic and related differences existing among regions.

The result of this study is also lower than a study conducted in refugee settings in Uganda (52.2%), Germany (47%), Eastern Nepal (75%), and Cameroon (75%) [9, 25–27]. The discrepancy might be the difference in the study population and sample size. This study includes only married women and does not include women living in union or unmarried women. This may underestimate the magnitude of unmet needs.

The results of this study align with previous research conducted in Kampala (39%) and Tigray refugee camps, where women of reproductive age reported a similar prevalence of unmet contraceptive needs (41.8%) [17, 28]. This similarity could be attributed to comparable study designs, the shared context of displacement among both populations, and a lack of emphasis from local health authorities on addressing the unmet demand for modern contraceptive methods.

In comparison to studies conducted in various locations for the general population, the prevalence of unmet contraceptive needs in this study was higher than that reported in the United States (12.1%), Burundi (32.4%), Hargeisa (30%), Mekelle City (19.7%), Gonji Kolela District (23.8%), Damot Woyde District (26.3%), and Dangila Town (17.4%) [22, 23, 29–33]. Additionally, it exceeded both the national (22%) and Oromia regional state (29%) total unmet needs for the general population [34]. This disparity may stem from limited access to FP services and a constrained supply of modern contraceptive methods in displaced settings, which restricts their use.

The study findings indicated a significant connection between the availability of modern contraceptive methods and the unmet need for these methods. The data suggested that participants with inadequate access to modern contraceptive methods were 1.738 times more likely to experience unmet needs compared to those with sufficient access to modern contraceptives. The finding of this study is in line with studies conducted among refugee women in Nigeria and Pakistan [9, 35]. They suggested that “there was a shortage of availability to utilize modern contraceptive methods; none of the consumers was using long and permanent modern contraceptive methods. Similarly, female condom utilization was almost zero, emergency contraception was only available in the condition of post-rape care; only short-term contraceptive methods were reported.” This also indicates that in a place where there are no availabilities of diverse methods of modern contraceptive methods, women did not get the desired methods and did not utilize undesired methods. This might be the reason why most refugees and IDPs set unmet needs for modern contraceptive methods very high.

This study, also in line with contraceptive availability, leads to an increase in use in the conflict-affected DRC: evidence from cross-sectional cluster surveys, facility assessments, and service statistics [36]. They suggested that demand for contraception, including long-acting methods, is present even in humanitarian settings and that women will use them when they are available and of reasonable quality. This finding is also supported by a study conducted in Tigray refugee camps and the multicountry baseline assessment done among women in selected refugee settings [17, 37]. The study conducted in Tigray refugee camps suggested that the odds of unmet need were 2.77 times higher among women who did not have enough availability of modern contraceptive methods as contrasted to women who had enough availability of modern contraceptive methods.

During the day of the survey, facility observation was done on the availabilities of modern contraceptives in a health center that gave service to this population. The observation showed that male condoms, female condoms, IUCD, and emergency contraceptives were completely unavailable. A shortage of oral contraceptive pills and injectables was reported by healthcare providers.

The research also found a significant correlation between the age at first marriage and the overall unmet need for modern contraceptive methods. Participants who married before the age of 18 were 1.559 times more likely to experience unmet needs compared to those who married at the age of 18 or older. This finding aligns with a study conducted in Ethiopia, including systematic reviews and meta-analyses in Shire-Enda-Selassie, Shebedino District, and Enemy District [38–41]. This might be due to younger women not having better awareness and information about FP, and they are also mentally and physically not mature enough to decide the number of children they desire related to women who joined marriage at the age of 18 years and above.

Respondents who did not have discussions about FP with healthcare providers were 1.673 times more likely to have an unmet need for modern contraceptive methods compared to those who did have these discussions. This finding aligns with a study conducted in Shire-Enda-Selassie [39]. This might be due to discussions with healthcare providers, providing women with information and knowledge about limiting and spacing the number of children they desire.

The study also found a significant association between the accessibility of FP services and the unmet need for modern contraceptive methods. Participants who had to travel for more than 30 min to access FP services were 1.551 times more likely to have an unmet need compared to those who traveled for less than 30 min. This finding is consistent with research conducted in refugee settings in Africa and Indonesia [14, 42]. The lack of comprehensive FP services in these settings, coupled with the low socioeconomic status of many women due to social breakdown, may hinder their access to FP services. Limited resources to pay for transportation and the absence of nearby facilities contribute to the challenges faced by women in using contraceptive methods. As a result, many women have to travel long distances on foot to access FP services.

### 4.1. Limitation of the Study

The study lacks triangulation from a qualitative study, which could have strengthened its findings. Additionally, it does not consider displaced unmarried women of reproductive age, as gathering information from this group was challenging. Furthermore, the study does not include the perspectives of male partners.

### 4.2. Strength of the Study

The modified version of the unmet need determination algorithm was used to demonstrate the level of total unmet need understandably. High response rates, consistent and standardized data collection protocols, and interviewer training may mitigate the impact of biases.

## 5. Conclusion and Recommendations

The study found a higher unmet need for spacing (35.4%) compared to limiting (5.3%). Factors such as accessibility of FP services, age at marriage, availability of modern contraceptive methods, and discussions with healthcare providers were significantly associated with the unmet need for modern contraceptives. The study suggests enforcing laws on minimum age at first marriage, educating women about contraception, and including FP topics in formal education. It also recommends strengthening the availability of modern contraceptives at displaced camps, improving discussions about FP with healthcare providers, and constructing comprehensive health centers with well-trained staff. The study calls for increased efforts by the West Arsi Zone Health Bureau and other sectors to reduce the high unmet need for modern contraceptives. Additionally, it recommends further research on factors such as fertility intentions, couples' unmet needs, and cultural and behavioral factors.

## Figures and Tables

**Table 1:** Sociodemographic characteristics of internally displaced currently married reproductive-age women in Bishan Guracha Town, West Arsi Zone (*n* = 393), 2021.

**Variables**	**Frequency**	**Percent**
Age of respondents (in years)		
15–24	58	14.8
25–34	271	69.0
35–49	64	16.3
Place of the previous residence		
Urban	253	64.4
Rural	140	35.6
Religion		
Muslim	356	90.6
Orthodox	11	2.8
Protestant	26	6.6
Ethnicity		
Oromo	379	96.4
Amhara	8	2.0
Educational status of respondents		
No education	166	42.2
Primary education	189	48.1
Secondary and above	38	9.7
Educational status of husband		
No education	84	21.4
Primary education	209	53.2
Secondary and above	100	25.4
Occupation of respondents		
Housewife	353	89.8
Daily laborer	13	3.3
Merchant	27	6.9
Monthly income level		
<1500 ETB	319	81.2
≥1500 ETB	74	18.8

*Note:* Other—Wolaita 6 (1.5%).

Abbreviation: ETB, Ethiopian Birr.

**Table 2:** Reproductive health characteristics of internally displaced currently married reproductive-age women in Bishan Guracha Town, West Arsi Zone (*n* = 393), 2021.

**Variables**	**Frequency**	**Percent**
Number of living children (in number)		
0	8	2.0
1–2	152	38.7
3–4	140	35.6
≥5	93	76.3
Age at first marriage (in years)		
<18	200	50.9
≥18	193	49.1
History of induced abortion		
Yes	87	22.1
No	306	77.9
Currently pregnant		
Yes	121	30.8
No	272	69.2
Current pregnancy status		
Intended	89	73.6
Mistimed	30	24.8
Unwanted	2	1.7
Last birth status		
Wanted	357	92.0
Mistimed	29	7.5
Unwanted	2	0.5
Future intention of the respondent to use modern contraceptive methods		
No need	296	75.3
Need for spacing	80	20.4
Need for limiting	17	4.3

**Table 3:** Family planning program and behavioral-related characteristics of internally displaced currently married reproductive-age women in Bishan Guracha Town, West Arsi Zone (*n* = 393), 2021.

**Variables**	**Frequency**	**Percent**
Distance to nearby health facility (in minute)		
<30	219	55.7
≥30	174	44.3
Availabilities of desired modern contraceptive methods		
Yes	166	42.2
No	227	57.8
Contraceptive ever use		
Yes	281	71.5
No	112	28.5
Discussion about FP use with their partners		
Yes	178	45.3
No	215	54.7
Availability of radio in the house		
Yes	155	39.4
No	238	60.6
Discussion about FP use with healthcare providers		
Yes	165	42.0
No	228	58.0

**Table 4:** Knowledge internally displaced currently married reproductive-age women in Bishan Guracha Town, West Arsi Zone (*n* = 393), 2021.

**Variables**	**Frequency**	**Percent**
Ever heard of modern contraceptive methods		
Yes	380	96.7
No	13	3.3
Type of modern contraceptive method you know		
Pills	289	73.5
Implants	254	64.5
Injectable	341	86.8
IUCD	102	26.0
Condom	79	20.1
Female sterilization	28	7.1
Male sterilization	25	6.4
Spermicidal	14	3.6
Source of information for modern contraceptive methods		
Health worker	313	79.6
Radio	280	71.2
Television	246	62.6
Friends	227	57.8
Social media	123	31.3
Newspapers	99	25.2
Advantages of modern contraceptive methods you know		
To delay pregnancy	366	93.1
To avoid unwanted pregnancy	194	49.4
To limit family size	146	37.2
For regulation of period	80	20.4
To prevent STI	61	15.5
Main places you know to get modern contraceptive methods		
Health center	385	98.0
Hospital	211	53.7
Health station	82	20.9
Shop and FGAE clinic	31	7.9
Private clinic	37	9.4
Pharmacy	103	26.2
Do you think that the use contraceptives is important		
No	79	20.1
Yes	314	79.9
Do you recommend using contraceptives for others		
No	89	22.6
Yes	304	77.4
Do you think it is possible to obtain this		
No	47	12.0
Yes	346	88.0
Knowledge status of respondents		
Good	123	31
Poor	270	69
Attitudinal status of respondents		
Favorable	189	48
Unfavorable	204	52

**Table 5:** Bivariate and multivariate logistic regression analyses of factors associated with unmet need for modern contraceptive methods among of internally displaced currently married reproductive-age women in Bishan Guracha Town, West Arsi Zone (*n* = 393), 2021.

**Variables**	**Unmet need for modern contraceptive methods**		**COR (95% CI)**	**AOR (95% CI)**
	**Yes**	**No**		
	**N** ** (%)**	**N** ** (%)**		
Age at marriage (in years)				
<18	93 (46.5)	107 (53.5)	1.635 (1.089–2.454)^∗^	1.559 (1.019–2.385)^∗^
≥18	67 (34.7)	126 (65.3)	1	1
The time taken to travel to the source of contraceptive methods (in minutes)				
≥30	86 (49.4)	88 (50.6)	1.915 (1.273–2.881)^∗∗^	1.551 (1.002–2.401)^∗^
<30	74 (33.8)	145 (66.2)	1	1
Discussions with healthcare providers				
Yes	79 (47.9)	86 (52.1)	1	1
No	81 (35.5)	147 (64.5)	1.667 (1.108–2.508)^∗^	1.673 (1.085–2.581)^∗^
Ever experienced abortion				
Yes	44 (50.6)	43 (49.4)	1.676 (1.038–2.707)^∗^	1.600 (0.966–2.651)
No	116 (37.9)	190 (62.1)	1	1
Knowledge of contraceptive methods				
Good	64 (52)	59 (48)	1	1
Poor	96 (35.6)	174 (64.4)	1.966 (1.276–3.031)^∗∗^	1.538 (0.964–2.454)
Availabilities of desired contraceptive methods				
No	104 (45.8)	123 (54.2)	1.661 (1.09–2.514)^∗^	1.738 (1.125–2.684)^∗^
Yes	56 (33.7)	110 (66.3)	1	1
Discussion with partners				
Yes	84 (47.2)	94 (52.8)	1	1
No	76 (35.3)	139 (64.7)	1.634 (1.089–2.453)^∗^	1.356 (0.833–2.207)
Attitude towards FP				
Favorable	84 (44.4)	105 (55.6)	0.742 (0.496–1.111)	0.941 (0.605–1.463)
Unfavorable	76 (37.3)	128 (62.7)	1	1

*Note:* 1.0 = reference category.

∗ and ∗∗ show the level of significance at *p* ≤0.05 and *p* ≤0.01, respectively.

## Data Availability

Data will be available without restriction.

## Nomenclature

AOR: adjusted odds ratio
COR: crude odds ratio
DRC: Democratic Republic of Congo
EC: Ethiopian calendar
EDHS: Ethiopian Demographic and Health Survey
FP: family planning
HEWs: health extension workers
HIV: human immunodeficiency virus
IAWG: Interagency Working Group
IDP: internally displaced person
IRB: Institutional Ethical Review Board
IUDs: intrauterine device
NGOs: nongovernmental organization
NRR: none response rate
RH: reproductive health
SDGs: sustainable development goals
SPSS: Statistical Package for Social Science
SRH: sexual and reproductive health
STI: sexually transmitted infection
UNHCR: United Nations High Commissioner for Refugees
VIF: variance inflation factor
