# Corrigendum to “Unmet Need for Modern Contraceptive Methods Among Displaced Married Women in Their Reproductive Years in Bishan Guracha Town, West Arsi Zone, Oromia Region, Ethiopia”

**DOI:** 10.1155/irem/9816815

**Published:** 2025-07-02

S. Million, Z. Gebru, S. Hassen, and S. Tesfaye, “Unmet Need for Modern Contraceptive Methods Among Displaced Married Women in Their Reproductive Years in Bishan Guracha Town, West Arsi Zone, Oromia Region, Ethiopia” *International Journal of Reproductive Medicine* 2024, no. 1 (2024): 1-11, https://doi.org/10.1155/2024/6662117.

In the article titled “Unmet Need for Modern Contraceptive Methods Among Displaced Married Women in Their Reproductive Years in Bishan Guracha Town, West Arsi Zone, Oromia”, there was a spelling error in the author list: Sultan Hassen should have been listed as Sultan Hussen Hebo. This has been corrected as shown below.

Sisay Million, Zeleke Gebru, Sultan Hussen Hebo, and Selamnesh Tesfaye

We apologize for this error.

